# The Impact of Depression and Leisure Activities on E-Health Literacy Among Older Adults: A Cross-Cultural Study in the EU and Japan

**DOI:** 10.3390/ijerph22030403

**Published:** 2025-03-10

**Authors:** Kumi Morishita-Suzuki, Toshimi Ogawa, Roberta Bevilacqua, Sebastien Dacunha, Vera Stara, Johanna Möller, Cecilia Palmier, Asako Ohara, Ai Abe, Denilson Brilliant T., Maribel Pino, Rainer Wieching, Elvira Maranesi, Anne-Sophie Rigaud, Shuichiro Watanabe, Volker Wulf, Yasuyuki Taki

**Affiliations:** 1 Sendai Center for Dementia Care Research and Practices, Sendai 989-3201, Japan; 2 Graduate School of Gerontology, J.F. Oberlin University, Tokyo 194-0213, Japan; swan@obirin.ac.jp; 3 Smart-Aging Research Center, Tohoku University, Sendai 980-8575, Japand.brilliantt@dc.tohoku.ac.jp (D.B.T.); yasuyuki.taki.c7@tohoku.ac.jp (Y.T.); 4 Scientific Direction, IRCCS INRCA, 60124 Ancona, Italy; r.bevilacqua@inrca.it (R.B.); v.stara@inrca.it (V.S.); e.maranesi@inrca.it (E.M.); 5 Maladie d’Alzheimer, Université de Parisance, 75006 Paris, France; sebastien.dacunha@aphp.fr (S.D.); maribel.pino@aphp.fr (M.P.); anne-sophie.rigaud@aphp.fr (A.-S.R.); 6 Service de Gériatrie 1 & 2, Hôpital Broca, Assistance Publique—Hôpitaux de Paris, 75013 Paris, France; 7 Diöcesan Caritas Association for the Archdiocese of Cologne, D-50676 Cologne, Germany; johanna.moeller@caritasnet.de; 8 Centre Hospitalier Jacques Coeur, 18000 Bourges, France; cecilia.palmier@ch-bourges.fr; 9 Misawa Homes Institute, Research and Development Co., Ltd., Tokyo 168-0072, Japan; asako_ohara@home.misawa.co.jp; 10 Institute for New Media & Information Systems, University Siegen, D-57072 Siegen, Germany; rainer.wieching@uni-siegen.de (R.W.); volker.wulf@uni-siegen.de (V.W.); 11 Department of Aging Research and Geriatric Medicine, Institute of Development, Aging and Cancer, Tohoku University, Sendai 980-8575, Japan

**Keywords:** digital health, aging population, mental health, social engagement, e-ViTA

## Abstract

Health services through digital technologies (e-health) offer a promising solution, but some older adults may encounter difficulties in utilizing these resources due to varying levels of e-health literacy. This study investigated the relationship between depression, leisure activities, and e-health literacy among older adults in the EU and Japan using cross-sectional data from the e-ViTA baseline survey. Findings revealed that depression was negatively associated with e-health literacy in both regions (EU: β = −0.54, 95% confidence interval (CI): −0.79, −0.28; Japan: β= −0.24, 95%CI: −0.46, −0.02). Leisure activities were positively associated with e-health literacy only in the EU (β = 0.55, 95%CI: 0.23, 0.87). Moreover, leisure activities appeared to partially mediate the relationship between depression and e-health literacy in the EU group. These results highlight the need to address mental health issues and promote leisure activities to improve e-health literacy among older adults, emphasizing tailored interventions for different cultural contexts.

## 1. Introduction

The rapid growth of the aging population has led to an imbalance between older adults who require medical and long-term care services and the available medical and long-term care workforce [1,2]. This imbalance hampers the accessibility and availability of these services for older adults. In Japan, one of the most rapidly aging countries, the aging rate was 29.0% as of 2022 [3]. In the European Union (EU), the aging rate was 21.1% as of 2022 [4]. In this context, the World Health Organization (WHO) believes that enhancing health services through digital technologies (e-health) can allow more people to achieve better health and well-being [5]. Health services through e-health were defined as health services and information disseminated through the internet and related technologies [6].

The benefits of e-health use for older adults’ overall health status and health-related behaviors have been reported [7,8,9,10,11]. A systematic review highlighted that e-health use improves health literacy, communication with healthcare professionals, positive emotions, and self-care among older adults [10]. Additionally, e-health usage eliminates the need for physical travel or face-to-face interactions for medical and long-term care services; therefore, it is particularly beneficial for older adults, who are at greater risk of physical and mental frailty [9]. However, some older adults may have limited experience or familiarity with technology [12,13]. Factors associated with the affinity of e-health among older adults include socioeconomic status (gender, age, race, education attainment, income status), health status (self-rated health, medical condition, depression), internet usage habits, digital literacy, and e-health literacy [12,13,14].

E-health literacy was defined as the ability to seek, find, understand, and appraise health information from electronic sources [15,16]. Developing e-health literacy is a key indicator of affinity and usage of e-health in older adults [17]. Older adults’ e-health literacy is affected by not only socioeconomic status but also their health status and social networks [18,19,20,21]. Given the strong correlation between the health status and social networks of older adults [22,23], it is essential to consider approaches to improve e-health literacy that address both factors.

Depression is negatively associated with e-health usage among older adults [13]. However, the association between depression and e-health literacy among older adults is unclear. Many intervention studies for promoting older adults’ e-health literacy were based on the self-efficacy theory [24,25]. The self-efficacy theory posits that an individual’s belief in their ability to perform a specific behavior successfully plays a crucial role in behavior change [26]. Higher self-efficacy enhances motivation, persistence, and resilience in acquiring new skills or knowledge. Conversely, people with depression often experience lower motivation, impaired cognitive function, and reduced self-efficacy, which may hinder their ability to develop e-health literacy skills [27,28]. Therefore, it is hypothesized that depression suppresses e-health literacy.

Leisure activities provide physical and cognitive stimulation, which are beneficial for enhancing the overall health of older adults [29,30]. Additionally, leisure activities are associated with the maintenance of social networks [31]. Leisure activities represent a broad concept, encompassing both social and solitary activities, such as watching movies or gardening [31]. Social networks are positively associated with e-health literacy in older adults [19]. Since leisure activities are an important component of social networks, they may also contribute to improving e-health literacy. Moreover, leisure activities are influenced by psychological factors, including depression. However, to our knowledge, the mediation effect of leisure activities between depression and e-health literacy has not been identified.

As previously mentioned, despite evidence that mental health and social factors influence e-health literacy, no studies have incorporated them into a single model to examine their association with e-health literacy. Investigating these associations could facilitate the creation of comprehensive interventions to enhance e-health literacy in older adults. Moreover, research in this area across different countries is needed to better capture the diverse lifestyles of older adults and improve the generalizability of findings. However, the body of research in this area remains limited. Therefore, this study aimed to clarify the association between depression, leisure activity, and e-health literacy among older adults by multi-country survey data. Our research questions (RQs) were: (RQ1) Is depression negatively associated with e-health literacy? (RQ2) Are leisure activities positively associated with e-health literacy? (RQ3) Do leisure activities reduce the negative association between depression and e-health literacy?

## 2. Materials and Methods

### Study Design and Participants

We used the cross-sectional data from the European project e-ViTA baseline survey. This research project focuses on empowering older adults via a socio-technological support system, the e-ViTA virtual coach, in Europe and Japan. The target population is healthy community residents aged ≥65 years in France (Paris), Italy (Ancona), Germany (Cologne), and Japan (Tokyo, Miyagi). For more details on subject recruitment and program specifics, refer to [32,33]. The baseline data used in our analysis were collected before any coaching or other interventions were provided to the participants.

## 3. Measures

### 3.1. Outcome

The e-HEALS [15] and the Japanese version [34] were used to assess e-health literacy. The tool consists of eight items, including questions about whether individuals are aware of the health information available on the internet, know where to find useful health information online, and understand how to search for health information on the internet. All items are rated on a five-point Likert scale, with scores ranging from 1 (very inconsistent) to 5 (very consistent), with higher scores representing higher self-perceived e-health literacy.

### 3.2. Independent Variable

The 15-item Geriatric Depression Scale (GDS-15) [35] and the Japanese version [36] were used as screening tools to assess depression status. The GDS-15 is designed to screen for depressive symptoms in older adults, ranging from 0 to 4 categorized as normal and 5 to 15 indicating possible depression [35,36].

### 3.3. Mediator Variable

Leisure activities were assessed using the question, “Are you regularly engaged in any leisure activity?” with response options of “yes” or “no”. Participants described the content of their leisure activities freely.

### 3.4. Covariates and Subgroup Indicator

Gender (men/women) and age were included as covariates. The subgroups by country were divided into the EU (*n* = 51), including France (*n* = 13), Italy (*n* = 28), Germany (*n* = 10), and Japan (*n* = 71). In addition to these covariates, socio-economic status (SES) factors, such as education level and economic status, as well as medical conditions, physical function, and cognitive function, are likely related and may serve as potential confounders. However, due to limitations in the study design and sample size, it was not possible to adjust for these factors.

## 4. Statistical Analysis

Chi-square tests were performed for categorical variables, and Student’s *t*-tests were performed for continuous variables to examine the differences in participants’ characteristics between the EU and Japan groups. The free-text responses regarding the content of leisure activities were categorized following Morrow-Howell N., et al. [37] and are presented in Supplementary Table S1 and S2.

**Figure 1:**
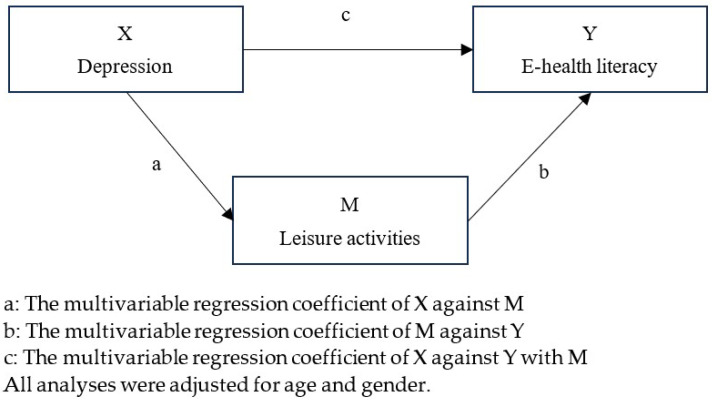
Illustration of the mediator analysis.

We used multivariable linear regression to analyze the main effect of depression and leisure activities on e-health literacy adjusted for gender and age (RQ1,2). Mediation analysis was performed based on Baron and Kenny [38] with multivariable linear regression (RQ3). Figure 1 shows the mediation analysis model. The mediation model is established if the following three conditions are met: (1) Depression is significantly associated with e-health literacy. (2) Depression is significantly associated with leisure activities. (3) When considering the associations of both depression and leisure activities with e-health literacy, the mediator, leisure activities, is significantly associated with e-health literacy, and the association between depression and e-health literacy either becomes non-significant or is reduced in strength compared to the direct association found in condition 1. In this mediation model, multivariate regression adjusted for age and gender was conducted. The mediation proportion (MP), defined as the dimensionless proportion of the effect of the independent variable on the dependent variable that is mediated through the mediator, was calculated following a previous study [39]. Additionally, we calculated point estimates and bias-corrected 95% confidence intervals for indirect effects using the bootstrap method with a sample size of 2000.

All analyses were conducted by EU and Japan subgroups due to potential cultural, social, and healthcare context differences that may influence the relationship between depressive symptoms, leisure activities, and e-health literacy. Statistical significance was set at *p* < 0.05. SPSS version 29.0 (IBM Corporation, Armonk, NY, USA) and Mplus version 8.10 were used for statistical analyses.

## 5. Results

### 5.1. Participants’ Characteristics

Table 1 shows the participants’ characteristics. The Japanese group had significantly more men and better e-health literacy scores than the EU group. The Supplementary Materials reveal the content of the leisure activities. Implementation rates were generally higher in the EU group across most activity categories.

### 5.2. The Association Between Depression, Leisure Activities, and E-Health Literacy (RQ1,2)

Table 2 presents the association between depression, leisure activities, and e-health literacy. Depression was significantly associated with decreased e-health literacy in both subgroups. However, the positive association between leisure activities and e-health literacy was significant only in the EU group. The Japanese group had no significant association between 144 leisure activities and e-health literacy.

### 5.3. The Mediation Effect of Leisure Activities Between Depression and E-Health Literacy (RQ3)

**Figure 2:**
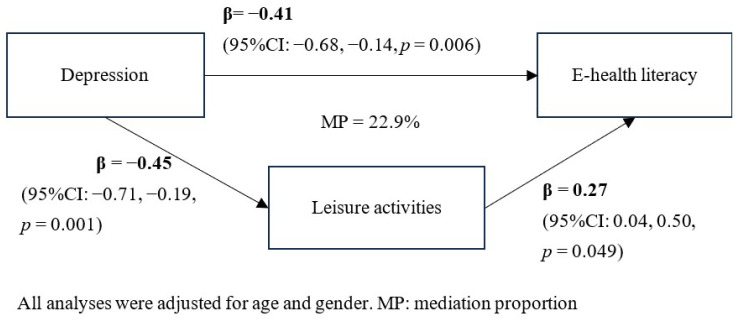
Mediation model in the EU group.

In the Japan group, the criteria of the aforementioned mediation model were not met, as there was no significant association between depression, leisure activities, and e-health literacy. Therefore, the mediation analysis was conducted only for the EU group. Figure 2 illustrates the output model for the mediation effect of leisure activities. The association between depression and leisure activities was significant. The increased leisure activities were significantly associated with higher e-health literacy. Additionally, the association between depression and e-health literacy (β= −0.41, 95% confidence interval (CI): −0.68, −0.14) weakens upon the addition of leisure activities to the main effect analyses in Table 2 (β= −0.54, 95%CI: −0.79, −0.28). The mediation effect of leisure activities was partial mediation whose MP was 22.9%. Additionally, the bootstrapped mediation model revealed a significant indirect effect of leisure activity on the relationship between depression and e-health literacy.

## 6. Discussion

We examined the association between depression, leisure activity, and e-health literacy among older adults across multiple countries, the EU and Japan groups. Our findings demonstrated that depression is negatively associated with e-health literacy in both the EU and Japan groups. Leisure activities show a positive association with e-health literacy, but this relationship was significant only in the EU group. Additionally, leisure activities partially mediated the relationship between depression and e-health literacy in the EU group.

Firstly, RQ1 posited that depression would be negatively associated with e-health literacy. Our findings supported this hypothesis in both the EU and Japan subgroups. This finding complements a previous study showing that depression is negatively associated with e-health usage among older adults [13]. Addressing depression could therefore be crucial in improving e-health literacy in this demographic.

Secondly, RQ2 posited that leisure activities would be positively associated with e-health literacy. Our findings supported this hypothesis in the EU subgroup. This finding reinforces a prior study [19] that found associations between social networks and e-health literacy. In Europe, engaging in leisure activities may enhance e-health literacy by providing opportunities for social interaction and cognitive stimulation. However, it is important to note that the leisure activities measure used in this study did not fully capture all aspects of social networks, such as the attributes and relationships of the individuals involved. Future analyses could further investigate the specific components of leisure activities that relate to e-health literacy and also examine which types of leisure activities may be more effective.

In RQ2, the Japan group showed no significant association between leisure activities and e-health literacy. Two things may explain this. (1) There are differences in how leisure activities are perceived. As shown in the Supplementary Materials, there is a considerable variation in the types of leisure activities undertaken, particularly in personal leisure and exterior household chores. Japanese individuals may view household chores as a duty rather than leisure, thus not including them in their leisure activities. This perception could lead to overlooking the potential social interactions with family members that accompany household chores. Additionally, many older adults in Japan receive familial support for using digital devices [40]. Moving forward, it will be important to clarify the definition of leisure activities and consider the role of interpersonal interactions. (2) Computer use, considered in leisure activities, was slightly more prevalent in the EU group. This factor could have influenced the outcomes since individuals who use computers often exhibit higher levels of e-health literacy [41].

Thirdly, RQ3 explored whether leisure activities mediate the relationship between depression and e-health literacy. The mediation analysis revealed that in the EU subgroup, leisure activities partially mediated this relationship. This suggests that engaging in leisure activities may be associated with a reduction in the negative relationship between depression and e-health literacy, possibly through enhanced social networks. In the future, it is crucial to acknowledge the impact of depression on e-health literacy and to develop targeted interventions for older adults affected by depression. Examples include integrating mental health support services into leisure programs, establishing counseling and peer support groups, and promoting activities that enhance psychological well-being. These efforts are essential for enhancing older adults’ e-health literacy by nurturing their social networks and overall well-being.

The study has several limitations. Firstly, the cross-sectional design prevents us from establishing causal relationships between the variables. Secondly, reliance on self-reported measures may introduce bias, as participants might overestimate or underestimate their e-health literacy and engagement in leisure activities. Thirdly, the generalizability of the findings is limited by the specific demographic and geographic characteristics of the sample, particularly the differences observed between the European and Japanese subgroups. Fourthly, in addition to the covariates included in the analysis, SES factors, such as education level and economic status, as well as medical conditions, physical function, and cognitive function, are likely related and could serve as potential confounders [20,21]. However, due to limitations in the study design and sample size, it was not possible to adjust for these factors. Future studies should aim to incorporate these additional variables to better control for potential confounding and provide a more comprehensive understanding of the factors influencing e-health literacy.

Despite these limitations, the study has notable strengths. It is among the few that investigate the interplay between depression, leisure activities, and e-health literacy across different cultural contexts. The use of a multi-country sample enhances the robustness of the findings and provides valuable insights into the diverse factors influencing e-health literacy among older adults. Additionally, the use of rigorous statistical methods, including mediation analysis and bootstrap techniques, strengthens the validity of the results.

## 7. Conclusions

This study suggests that older adults with depressive symptoms tend to have lower e-health literacy among both EU and Japanese participants. However, in the EU subgroup, engaging in leisure activities may be associated with a weaker negative relationship between depressive symptoms and e-health literacy. Future research should explore the underlying mechanisms of this association and examine whether interventions promoting leisure activities could contribute to better e-health literacy among older adults. Additionally, longitudinal studies are needed to clarify the causal relationships between depressive symptoms, leisure activities, and e-health literacy. Furthermore, to better understand the differences in results between the EU and Japan, future studies should expand the sample size and conduct more comprehensive analyses.

## Figures and Tables

**Table 1:** Participants’ characteristics.

		EU (*n* = 51)	Japan (*n* = 71)	*p*
Age				
	Mean ± SD	75.2 ± 7.0	73.5 ± 6.3	0.153
Gender				
	Men	37.3%	59.2%	0.017
	Women	62.7%	40.8%	
Depression				
	No	68.6%	83.1%	0.061
	Yes	31.4%	16.9%	
Leisure activities				
	No	17.6%	25.4%	0.312
	Yes	82.4%	74.6%	
E-health literacy				
	Mean ± SD	27.7 ± 12.5	35.1 ± 9.0	<0.001

Differences between the EU and Japan were evaluated using the Chi-square tests and Student’s *t*-tests. SD: standard deviation.

**Table 2:** The association between depression, leisure activities, and e-health literacy.

	EU (*n* = 51)						Japan (*n* = 71)					
	E-Health Literacy			E-Health Literacy			E-Health Literacy			E-Health Literacy		
	β	(95%CI)	*p*	β	(95%CI)	*p*	β	(95%CI)	*p*	β	(95%CI)	*p*
Depression												
Yes (Ref. No)	−0.54	(−0.79, −0.28)	<0.001	-	-	-	−0.24	(−0.46, −0.02)	0.03	-	-	-
Leisure activities												
Yes (Ref. No)	-	-	-	0.55	(0.23, 0.87)	0.001	-	-	-	0.11	(−0.08, 0.30)	0.24

95%CI: 95% confidence interval. All analyses were adjusted for age and gender.

## Data Availability

Data sharing is not applicable to this article.

## Supplementary Materials

The following supporting information can be downloaded at: https://www.mdpi.com/article/10.3390/ijerph22030403/s1, Table S1: The content of leisure activities.; Table S2: Categories of Leisure Activities According to Morrow-Howell et al.
