# Supplementary material for: The Impact of Depression and Leisure Activities on E-Health Literacy Among Older Adults: A Cross-Cultural Study in the EU and Japan
Source: Int J Environ Res Public Health. 2025 Mar 10;22(3):403. doi: 10.3390/ijerph22030403 (PMC11941757; doi:10.3390/ijerph22030403)
Supplement: Supplementary file 1 [file ijerph-22-00403-s001.zip › ijerph-3466064-supplementary.pdf]

Supplementary Table

Table S1. The content of leisure activities.

	EU (n = 42)	Japan (n = 53)
Personal leisure	26.2%	3.8%
Civic/religious activity	40.5%	41.5%
Physical exercise	57.1%	30.2%
Interior household chores	9.5%	7.5%
Exterior household chores	21.4%	7.5%
Computer use	7.1%	1.9%
Interpersonal exchange/helping others	9.5%	9.4%
Community leisure	9.5%	1.9%

Table S2. Categories of Leisure Activities According to Morrow-Howell et al.

Categories	Items
Personal leisure	Watch TV
	**Read papers/magazines/books**
	Play cards/games/puzzles
Civic/religious activity	Pray/meditate
	**Volunteering**
	**Religious attendance**
	**Attend meetings**
	**Sing/play instruments**
Physical exercise	Listen music
	**Walk**
	**Sport/exercise**
Interior household chores	House cleaning
	Wash/iron/mend
	Shop/run errands
	**Meal preparation/clean-up**
	Money management
	**Arts and crafts**
Exterior household chores	**Yard work/garden**
	**Pet care**
	Home improvement
	Vehicle maintenance
Managing medical conditions	Managing medical conditions
	Seeing a physician/nurse/and so on
	Managing medical bills
Employment/computer use	**Use computer**
	Employment
Interpersonal exchange/helping others	**Visit in person**
	Phone/letter/E-mail
	Show affection
	**Help others**
	Treating others' medical condition
Community leisure	**Concert/movies/lectures**
	**Leisure dining/eat out**

Bold items were answered by this study's participants.
